# Local and systemic signaling of iron status and its interactions with homeostasis of other essential elements

**DOI:** 10.3389/fpls.2015.00716

**Published:** 2015-09-14

**Authors:** Sheena R. Gayomba, Zhiyang Zhai, Ha-il Jung, Olena K. Vatamaniuk

**Affiliations:** Soil and Crop Sciences Section, School of Integrative Plant Sciences, Cornell University, IthacaNY, USA

**Keywords:** *Arabidopsis thaliana*, iron homeostasis, iron transporters, iron signaling, iron ligands, oligopeptide transporters

## Abstract

Iron (Fe) is essential for plant growth and development. However, alkaline soils, which occupy approximately 30% of the world’s arable lands, are considered Fe-limiting for plant growth because insoluble Fe (III) chelates prevail under these conditions. In contrast, high bioavailability of Fe in acidic soils can be toxic to plants due to the ability of Fe ions to promote oxidative stress. Therefore, plants have evolved sophisticated mechanisms to sense and respond to the fluctuation of Fe availability in the immediate environment and to the needs of developing shoot tissues to preclude deficiency while avoiding toxicity. In this review, we focus on recent advances in our understanding of local and systemic signaling of Fe status with emphasis on the contribution of Fe, its interaction with other metals and metal ligands in triggering molecular responses that regulate Fe uptake and partitioning in the plant body.

## Introduction

Iron (Fe) is essential for growth and development of all organisms, but can be toxic to cells in excess. The essential, yet toxic nature of Fe results from its ability to change the oxidation state (Fe^3+^ ↔ Fe^2+^; Marschner, 1995). Therefore, Fe participates in electron transfer reactions and is central to the function of heme- and Fe–S cluster-requiring enzymes participating in biological processes that include respiration, photosynthesis, sulfur assimilation, and nitrogen fixation (Marschner, 1995). However, non-coordinated Fe ions catalyze the formation of reactive oxygen species *via* Fenton reaction and might mismetallate essential cellular molecules, further exacerbating cellular damage (Valko et al., 2005). Therefore, internal Fe ions not associated with metalloenzymes are distributed among low molecular weight (LMW) ligands and constitute the labile (*alias* bioavailable) Fe pool. In this form, Fe is available for equilibration with metalloregulators and metalloenzymes *via* ligand exchange reactions to perform essential cellular functions (Hider and Kong, 2013). The concentration of the cytosolic labile Fe pool is defined in accordance with Fe needs of the plant through the ability of plants to sense changes in Fe bioavailability in the rhizosphere, in the cytosol and in the developing shoot, and integrate local and long-distance signaling events into a concerted action of Fe transporters and their regulators. Evidence of local and systemic signaling networks and crosstalk between them have been accumulating for more than a decade and are based on characteristic root responses to Fe deficiency such as the increase of the root surface-associated proton extrusion activity and the transcriptional responses of the Fe uptake system. As exemplified by studies in *Arabidopsis thaiana, IRT1* (**I**ron-**R**egulated **T**ransporter 1, encoding a high-affinity Fe(II) transporter) and *FRO2* (**F**erric **R**eduction **O**xidase 2, encoding a ferric chelate reductase) are transcriptionally regulated in response to local and long-distance signals of Fe availability, and these types of proteins constitute the major entry point of Fe into root epidermal cells in non-gramenaceous plants (reviewed in, Hindt and Guerinot, 2012; Kobayashi and Nishizawa, 2012).

Regulation of Fe uptake by locally originated signals have been observed in potato *(Solanum tuberosum*) by Bienfait et al. (1985). Roots of potato growing on low Fe nutrient solution exhibit increased proton extrusion and ferric reductase activity with, as well as without the sprout, pointing to the existence of a local, shoot-independent Fe deficiency signaling network in the root. Local Fe supply also triggers lateral root elongation in *Arabidopsis thaliana* (Giehl et al., 2012). On the other hand, ferric chelate reductase activity in roots of pea (*Pisum sativum*) fluctuates during the life cycle even when plants are grown under continues Fe supply, and is lowered when phloem transport to roots is interrupted (Grusak, 1995). This result suggested the existence of a shoot-borne signal that communicates Fe demands to the root. The latter hypothesis was further substantiated by results from experiments using the split-root system in *Plantago lanceolata* and *A. thaliana* showing that ferric chelate reductase activity increases in the half of the root grown with Fe, suggesting that shoots transmit an Fe-deficiency signal from the Fe-deficient to Fe-sufficient half of the root (Romera et al., 1992; Schmidt et al., 1996; Vert et al., 2003). Further, local and long-distance signals do not act independently from one another. Crosstalk between local and long-distance control of Fe deficiency response was shown by finding that while lateral root elongation in response to localized Fe supply is severely repressed in the *irt1* mutant of *A. thaliana* lacking functional Fe(II) transporter, the requirements for IRT1 is circumvented by the application of Fe to the shoot (Giehl et al., 2012).

These experiments raised many questions regarding the sites of signal(s) generation, the nature of local and shoot-borne signals, as well as sensors/receptors, which perceive and transduce information about local and long-distance Fe availability. Hormones, Fe-binding ligands, and recirculating Fe ions themselves have been proposed to act as local and long-range signals promoting Fe-deficiency responses in the root. Notably, fluctuation of cellular Fe concentration alters ratios of other transition elements and thus, has a profound effect on the metal composition of the cell (the metallome). Therefore, since many transition metals are close in their coordination geometry and ligand preferences (Haas and Franz, 2009), mismetallation of Fe-requiring apoenzymes and metalloregulators may occur under Fe deficient conditions, affecting Fe signaling and downstream transcriptional and posttranscriptional events. This review focuses mainly on the role of labile Fe, Fe ligands, and crosstalk among essential elements in local and systemic Fe status signaling. Involvement of other factors in Fe status signaling is reviewed in (Hindt and Guerinot, 2012; Kobayashi and Nishizawa, 2012, 2014).

## Overview of Fe uptake, Root-to-Shoot Partitioning, and Speciation in Plant Tissues

**FIGURE 1:**
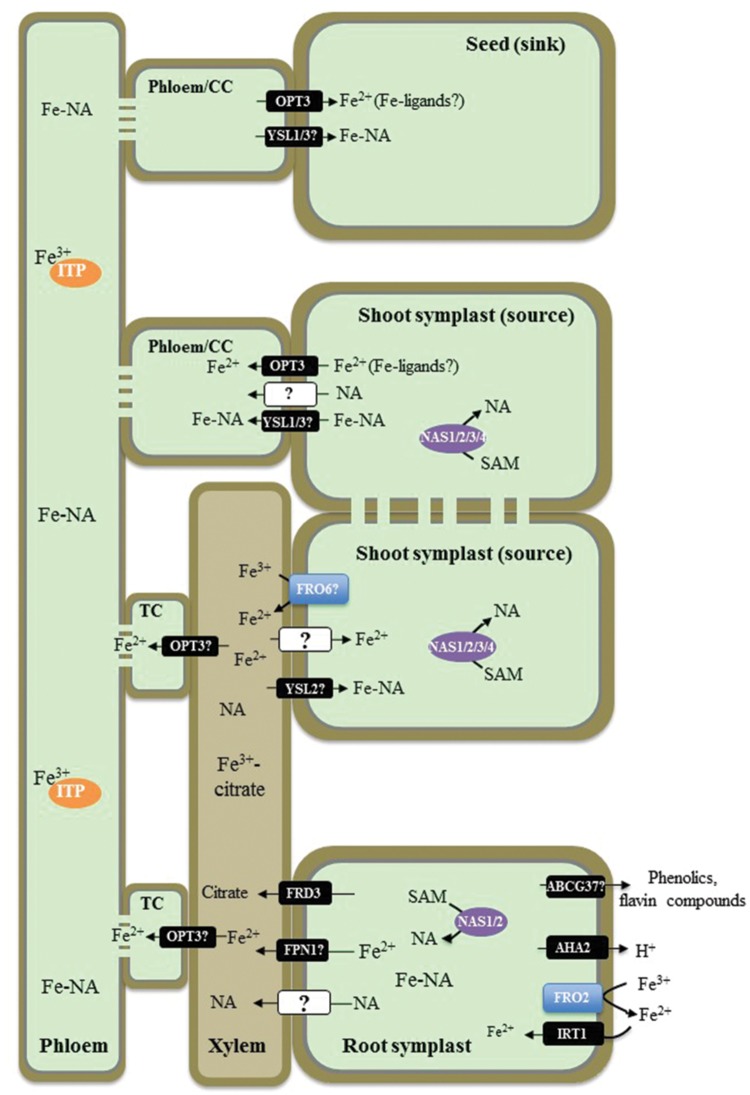
**Overview of iron (Fe) transport in non-graminaceous plants based on studies in *Arabidopsis thaliana*.** A proton pump, AHA2, contributes to the acidification of rhizosphere to facilitate solubilization of Fe(III) under Fe-limited conditions. Ferric-chelate reductase FRO2 reduces Fe(III) to Fe(II) at the root surface and Fe^2+^ is then transported into root epidermal cells by IRT1. Phenolic and flavin compounds are also exuded into the rhizosphere to facilitate Fe(III) solubilization by ABCG37/PDR9. Fe–NA chelates prevail in the cytosol, where nicotianamine (NA) in roots is produced by NASes. Fe–NA then moves radially via plasmodesmata toward the vasculature. FPN1 is implicated in loading Fe into the xylem vessels, while FRD3 provides citrate. In the xylem, Fe forms a (tri)Fe^3+^-(tri)citrate complex, which moves toward the shoot via the transpiration stream. In shoots Fe(III) is reduced likely by the action of the FRO family members. YSL2 is proposed to transport Fe–NA to photosynthetic cells (mesophyll cells). Whether the ionic form of Fe is reabsorbed from the xylem vessels into xylem parenchyma cells in the shoot, is not known. In the shoot symplast, Fe predominantly exists as Fe(II)–NA complex. Fe is then loaded into the phloem for subsequent partitioning to sink tissues such as young leaves, seeds as well as is recirculated to the root. OPT3 and YSL1 and YSL3 are implicated in Fe^2+^ and Fe–NA loading into the phloem, respectively. While Fe–NA is a predominant form of Fe in the phloem sap, Fe transporting protein (ITP), metallothionein-like protein type 2B, and a low-molecular weight protein with the similarity to a phloem-specific protein from *M. truncatula*, have been identified associated with Fe in the phloem sap of *R. communis* and *L. texensis*. CC, companion cells; TC, phloem companion cells that de-differentiated into transfer cells.

Our understanding of signaling events regulating Fe status in the cell is based on tracking several characteristic responses of Fe transport systems summarized below. To acquire Fe from the rhizosphere, non-graminaceous, and graminaceous plants use the reduction strategy (Strategy I) and the chelation strategy (Strategy II), respectively (Marschner and Romheld, 1994). For an overview of the chelation strategy, we would like to refer the reader to (Walker and Connolly, 2008; Hindt and Guerinot, 2012). This review will focus on the components of the Fe transport machinery in plants utilizing the reduction strategy (**Figure 1**). In brief, this strategy includes the acidification of the rhizosphere by root plasma membrane H^+^- ATPases, which increase the solubility of Fe(III) (Santi and Schmidt, 2009) and the reduction of Fe(III) chelates to soluble Fe(II) by the root plasma membrane ferric chelate reductase, FRO2 in *A. thaliana* and FRO1 in *Pisum sativum* (Robinson et al., 1999; Waters et al., 2002). The solubilized Fe(II) enters the apoplastic space where it constitutes up to 75% of the total Fe of the root (Bienfait et al., 1985). Although Fe can be remobilized from the apoplast into the symplast in any cell type of the root, the expression pattern of the key Fe transporter in *A. thaliana*, IRT1, suggests that the bulk of Fe(II) uptake into the symplast occurs in the root epidermal cells (Eide et al., 1996; Vert et al., 2002). The FRO2/IRT1-like system constitutes the major pathway for Fe entry into root epidermal cells of non-graminaceous plants. Given the essential and toxic nature of Fe, expression of genes encoding root ferric chelate reductases and *IRT1* is tightly regulated by Fe status and thus, has been used in studies of local and long-distance Fe status signaling (Hindt and Guerinot, 2012; Kobayashi and Nishizawa, 2012). A second “auxiliary” Fe uptake strategy involves the exudation of phenolic and flavin compounds into the rhizosphere during Fe deficiency to facilitate utilization of apoplastic Fe reserves (Bienfait et al., 1985; Jin et al., 2007; Lan et al., 2011; Rodríguez-Celma et al., 2011; Rodríguez-Celma and Schmidt, 2013; Schmid et al., 2014). Recent studies in grasses have shown that the protocatechuic acid eﬄuxer **P**henolics **E**ﬄux **Z**ero **1 (**PEZ1), is required for the mobilization of Fe in the stele of rice, and transgenic rice plants overexpressing *PEZ1* grow better on soils with high pH and poor Fe availability (Ishimaru et al., 2011). PEZ1 counterpart has not been yet identified in non-graminaceous plants. However, recent studies in *A. thaliana* indicated that an ATP-binding cassette transporter ABCG37 (*alias* PDR9) is involved in secretion of phenolic compounds from roots into the medium in response to Fe deficiency (Rodríguez-Celma et al., 2013; Fourcroy et al., 2014).

After entering root epidermal cells, Fe moves symplastically via plasmodesmata toward the vasculature and is eﬄuxed from xylem parenchyma cells into the xylem vessels where it is chelated by citrate to form a tri-Fe(III) tri-citrate complex to undergo long-distance transport to the shoot (Larbi et al., 2010; Rellan-Alvarez et al., 2010). The key identified players contributing to this process in *A. thaliana* are the **M**ultidrug **a**nd **T**oxin **E**ﬄux family (MATE) member, FRD3, and one of two members of the **I**ron **Reg**ulated1/**F**erro**p**orti**n** 1 family, IREG1/FPN1 (Green and Rogers, 2004; Durrett et al., 2007; Morrissey et al., 2009). FRD3 is located in the root pericycle (Rogers and Guerinot, 2002; Green and Rogers, 2004), transports citrate from xylem parenchyma cells into xylem vessels (Durrett et al., 2007) and acts in concert with IREG1/FPN1, which is proposed to mediate Fe eﬄux into xylem vessels (Morrissey et al., 2009). Citrate release into the apoplast *via* FRD3 plays an important role in partitioning Fe between the apoplast and symplast of cells surrounding xylem vessels in roots and leaf mesophyll cells (Roschzttardtz et al., 2011). The lack of proper long distance Fe transport in xylem vessels results in shoot chlorosis and deregulated Fe signaling in the *frd3* mutant (discussed below). A mutation in *IREG1/FPN1*, however, does not prevent Fe accumulation in the root vasculature suggesting that another Fe transporter(s) operates redundantly with IREG1/FPN1 (Morrissey et al., 2009). Similar to *frd3* mutants, *A. thaliana fpn1* mutant plants develop shoot chlorosis when grown in Fe-deficient media. However, the function of IRT1 and FRO2 in the *fpn1* mutant is not affected, indicating that Fe signaling is not altered with the loss of IREG1/FPN1 (Morrissey et al., 2009).

Next steps of Fe transport within the plant include the re-absorption of Fe from the apoplast of xylem vessels in the shoot for distribution to leaf parenchyma cells, apoplastic phloem loading for partitioning into sink tissues, and phloem-based recirculation to the root. The central transporters involved in these processes in *A. thaliana* belong to two distinct clades of the **O**ligo**p**eptide **T**ransporter (OPT) family: the **Y**ellow **S**tripe-**l**ike1 (YSL) proteins and OPTs, for which the family was named (DiDonato et al., 2004; Schaaf et al., 2005; Waters et al., 2006; Curie et al., 2009; Lubkowitz, 2011). YSLs are proposed to be involved in the long-distance transport of transition metals, primarily Fe, Cu, and Zn, associated with a strong metal ligand, nicotianamine (NA; DiDonato et al., 2004; Schaaf et al., 2005; Waters et al., 2006; Chu et al., 2010). Based on the expression of *AtYSL2* in xylem parenchyma cells and its involvement in Fe homeostasis in *A. thaliana*, YSL2 has been proposed to function in lateral Fe movement in the vasculature (DiDonato et al., 2004; Schaaf et al., 2005). The tissue expression studies and phenotypes of the single *ysl1* and the double *ysl1ysl3* mutant plants suggest that YSL1 and YSL3 play a redundant role in the delivery of Fe, as well as Cu and Zn, into seeds of *A. thaliana* (Waters et al., 2006; Chu et al., 2010). OPT3, a close relative of the YSLs and a member of the OPT family, contributes to Fe delivery to seeds and loss of this function is suggested to cause embryo lethality in the *opt3-1* null mutant (Stacey et al., 2002, 2008; Mendoza-Cozatl et al., 2014; Zhai et al., 2014). Knockdown *opt3* mutants are viable but accumulate high levels of Fe in both shoots and roots while exhibiting deregulated systemic Fe signaling marked by constitutive expression of *IRT1* and *FRO2* (Stacey et al., 2008; Mendoza-Cozatl et al., 2014; Zhai et al., 2014). Based on the ability of OPTs to transport peptides in heterologous systems (for review see, Lubkowitz, 2011), OPT3 has been suggested to transport Fe, chelated with a peptide-based ligand or Fe–NA complexes. It has been shown, however, that NA does not improve the ability of OPT3 to complement growth of Fe uptake deficient, the *fet3fet4* mutant of *S. cerevisiae* on Fe-limited medium, suggesting that OPT3 is involved in Fe transport and its contribution to Fe–NA transport has to be examined further (Wintz et al., 2003). Given that a ubiquitous tripeptide glutathione, GSH, is among the plausible OPT substrates, OPT3 was tested for the ability to transport GSH or Fe–GS complexes. However, regardless of whether studies were done in a heterologous system or *in planta*, OPT3 was not capable of transporting GSH (Mendoza-Cozatl et al., 2014; Zhai et al., 2014). Studies using *Xenopus* oocytes showed that OPT3 mediates uptake of Fe^2+^ and also Cd^2+^in a time-dependent manner in medium lacking metal ligands, suggesting that OPT3 is a transition metal transporter (Zhai et al., 2014). Future studies should test, however, if the addition of potential Fe and/or Cd ligands would alter this transport capability of OPT3 in oocytes. It is also important to determine whether OPT3 transports naked ions *in planta* as well, or is also capable to transport ion-ligand complexes. Concerning the physiological function of OPT3, it has been shown that it is expressed in the phloem where it is located to companion cells, and functions in Fe^2+^ partitioning from sources (mature leaves) to sinks such as young leaves and seeds (Mustroph et al., 2009; Mendoza-Cozatl et al., 2014; Zhai et al., 2014). Finally, data from Zhai et al. (2014) suggest that OPT3 contributes to Fe recycling from the xylem and acts as a functional link between the xylem and the phloem.

The cytosolic concentration of Fe is controlled mainly through Fe release from or import into the vacuole. The Mn/Fe exporters, NRAMP3 and NRAMP4 mediate retrieval of Fe from vacuoles and function of these transporters is particularly essential during germination (Thomine et al., 2003; Lanquar et al., 2005). **V**acuolar Iron **T**ransporter 1-**l**ike (VTL) proteins and a second ferroportin transporter in *A. thaliana*, FPN2, mediate Fe import into the vacuole (Kim et al., 2006; Morrissey et al., 2009; Gollhofer et al., 2014). **V**acuolar **I**ron **T**ransporter 1 (VIT1), is expressed in the vasculature of seedlings and developing seeds; *vit1-1* plants are unable to properly store Fe in seeds and embryos, resulting in reduced fitness of seedlings on soils with alkaline pH levels (Kim et al., 2006; Roschzttardtz et al., 2009).

The route of Fe from the rhizosphere into plant roots and Fe movement throughout the plant involves Fe(II) ↔ Fe(III) oxidation ↔ reduction steps and ligand exchange reactions. The predominant form of Fe in the rhizosphere, Fe(III), is reduced by ferric chelate reductases to Fe(II) prior entering the symplast in the root. Fe(II) must be then oxidized to Fe(III) in the xylem since the latter is a predominant oxidation state of Fe in the xylem sap (Larbi et al., 2010; Rellan-Alvarez et al., 2010). Fe(III) exists in the xylem sap predominantly as a tri-iron(III), tri-citrate complex (Rellan-Alvarez et al., 2010). Since the redox potential of the cytoplasm favors Fe(II) over Fe(III), it is likely that Fe(II) constitutes the labile Fe pool of the plant cell, including the cytoplasm of sieve elements in the phloem (Williams, 1982). Therefore, Fe(III) must be reduced before entering a symplast in the shoot and members of the FRO family might be responsible for this event. In this regard, *FRO6* is expressed at high level in leaves and reduces Fe(III) to Fe(II) at the cell surface in green tissues and thus, might be involved in Fe(III) to Fe(II) reduction prior loading of Fe(II) into leaf cells (Feng et al., 2006; Mukherjee et al., 2006; Jain et al., 2014). However, Grillet et al. (2014) have shown recently that ferric reduction activity is not responsible for Fe(III) to Fe(II) reduction in pea embryos, which absorb Fe(III) as a complex with citrate and malate from maternal tissues (Grillet et al., 2014). They found that the reduction step in embryos is absolutely dependent on the eﬄux of ascorbate. Further, this group showed that a similar, ascorbate-dependent Fe(III) to Fe(II) reduction occurs in embryos of *A. thaliana* (Grillet et al., 2014). Rellán-Álvarez et al. (2008) has shown that at pH 5.5 and 7.5, Fe-citrate undergoes exchange reactions with NA. This group suggested that at the more neutral pH of the cytosol and the phloem sap, the Fe(II)–NA complex prevails. In contrast, the tri-Fe(III) tri-citrate complex is the prevailing form of Fe in more acidic conditions in the xylem sap, apoplast, and the vacuole.

## Local and Systemic Signaling of Fe Status

### The Role of Fe Availability in Local and Systemic Signaling

Iron bioavailability has been suggested to play a dual role in signaling: it acts as a positive regulator in local Fe deficiency responses but as a negative regulator in systemic deficiency responses. With regard to local regulation, Vert et al. (2003) have suggested that the pool of available Fe in the root apoplast controls the expression of *IRT1* and *FRO2* in *A. thaliana.* They found that the low concentration of Fe in the apoplast caused by prolonged Fe deficiency stops transcript accumulation of *IRT1* and *FRO2*. This response is reversed when Fe-starved plants are resupplied with Fe. In addition to apoplastic regulation, the concentration of Fe in the cytosol regulates induction of *IRT1* and *FRO2*. In this regard, vacuolar membrane-localized NRAMP3 and IREG2/FPN2 influence *IRT1* and *FRO2* expression by controlling the vacuolar/cytosol Fe ratio in *A. thaliana* (Thomine et al., 2003; Lanquar et al., 2005). Thomine et al. (2003) showed that ectopic overexpression of *NRAMP3* downregulates the expression of *FRO2/IRT1* in *A. thaliana* likely due to enhanced cellular Fe availability via vacuolar Fe release (Thomine et al., 2003; Lanquar et al., 2005). Although results from ectopic overexpression may not reflect a physiological role of NRAMP3, this example is used to highlight the role of cytosolic Fe pools in the regulation of Fe deficiency responses in the root. Delayed induction of *IRT1* and lower ferric chelate reductase activity was reported in *fpn2-1* mutants, which lack the vacuolar transporter IREG2/FPN2 (Morrissey et al., 2009). The late response in mutant plants was suggested to be due to the excess of Fe in the cytoplasm (Morrissey et al., 2009).

With respect to long-distance signaling, it has been suggested that the concentration of Fe in the phloem might play a signaling role in the shoot-to-root communication of Fe demands (Maas et al., 1988). Maas et al. (1988) showed that phloem sap of Fe-deficient castor oil (*Ricinus communis*) has lower concentration of Fe and roots have increased proton extrusion and ferric reductase activities, which could be reversed by the application of Fe to the leaves or by the transfer of whole plants to Fe-containing nutrient solution (Maas et al., 1988). The authors suggested that the capacity of the phloem to carry Fe to the root is sufficient to influence its Fe deficiency responses; the authors proposed that the Fe-related signal plays a repressive role in the phloem (Maas et al., 1988). Significant progress in understanding the role of Fe in systemic Fe signaling has been made using *opt3-2* and *opt3-3* mutants of *A. thaliana* (Stacey et al., 2008; Mendoza-Cozatl et al., 2014; Zhai et al., 2014) and **Table 1**. Both mutant alleles accumulate high levels of Fe in shoots, while exhibiting constitutive Fe starvation phenotypes. Zhai et al. (2014) have shown that grafting of wild-type shoots onto *opt3-3* roots rescues the constitutive Fe deficiency phenotype of the root. Consistently, Mendoza-Cozatl et al. (2014) have found that ectopic expression of *OPT3* in the shoot of the *opt3-2* mutant complements the *opt3-2* mutant phenotype. Together, these studies indicate that OPT3 function in the shoot regulates Fe deficiency responses of the root. Studies using *Xenopus* oocytes showed that OPT3 mediates uptake of Fe^2+^ in a time-dependent manner in medium lacking metal ligands (Zhai et al., 2014). Consistent with the role of OPT3 in loading Fe into the phloem, Zhai et al. (2014) found that the concentration of Fe in the phloem sap of the *opt3-3* mutant is significantly lower than in the wild-type. Given that the *opt3-3* mutant overexpresses *IRT1* and *FRO2* in the root even under Fe-sufficient conditions, the lower Fe concentration in the phloem sap of the mutant substantiates the hypothesis that Fe concentration in the phloem plays an essential and inhibitory role in regulating Fe deficiency responses in the root. The inability of *opt3* mutants to load Fe into the phloem in leaves for the long-distance transport to sinks also explains the failure of foliar application of Fe to rescue the constitutive Fe deficiency responses of roots of *opt3* mutant alleles (Garcia et al., 2013). It is noteworthy that the concentration of Fe in the xylem of the *opt3-3* mutant is 40-fold higher than in the xylem of wild-type, suggesting that OPT3 mediates Fe recirculation from the xylem to the phloem via xylem-to-phloem transfer for subsequent phloem-based partitioning into sinks (Zhai et al., 2014).

**Table 1:** Description of mutant alleles exhibiting de-regulated iron (Fe) signaling.

Organism/ Gene (allele)	Function	Cellular localization/tissue expression	Expression of *FRO2/IRT1*	Fe concentration in the mutant vs. wild-type	Treatments that rescue Fe deficiency symptoms	Reference
*Arabidopsis thaliana /FRD3* *(frd3)*	Citrate eﬄux	Plasma membrane/ Root pericycle	Constitutive expression of *FRO2* and *IRT1*	High Fe accumulation in apoplast and vasculature, but less Fe in cytosol	Foliar application of Fe; grafting *frd3* shoots onto Wt roots; citrate addition to growth media	Rogers and Guerinot, 2002; Green and Rogers, 2004; Garcia et al., 2013
*A. thaliana* */NAS1, NAS2* *NAS3, NAS4* *(nas4x-1, nas4x-2)*	nicotianamine (NA) synthesis	Cytosol/ Roots (*NAS1/2*) and shoots (*NAS1/2/3/4*)	Elevated expression of *FRO2, IRT1*, and *FIT* in Fe-sufficient plants in the *nas4x-1*	Elevated Fe concentration in the whole rosette of the *nas4x-1* mutant compared at the reproductive but not at the vegetative stage. Higher Fe in older leaves vs. younger leaves in the *nas4x-1 mutant*	Foliar application of Fe or NA rescues interveinal leaf chlorosis of *nas4x-2*	Bauer et al., 2004; Klatte et al., 2009; Schuler et al., 2012
*A. thaliana* */OPT3* *(opt3-2* and *opt3-3)*	Fe influx	Plasma membrane/ Phloem companion cells	Constitutive expression of *FRO2* and *IRT1*	High Fe accumulation in shoots Higher Fe in older leaves vs. younger leaves	Grafting Wt shoots onto *opt3* roots Expression of *OPT3* in leaves of the *opt3-2*	Stacey et al., 2008; Mendoza-Cozatl et al., 2014; Zhai et al., 2014
*A. thaliana* */YSL1, YSL2, YSL3(ysl1,ysl2 ysl1ysl3)*	Fe–NA transport	Plasma membrane/ Vascular parenchyma cells (YSL1 and YSL3), xylem parenchyma cells in roots (YSL2)	Lower or wild-type FRO2 activity in *ysl1ysl3* mutants, not tested in *ysl1* or *ysl2* mutants	No difference in *ysl2-1* mutant Less Fe in seeds of *ysl1* single mutant Less Fe in roots and shoots of *ysl1ysl3* double mutants	Foliar application of Fe increasing Fe in growth media for detached roots	DiDonato et al., 2004; Jean et al., 2005; Waters et al., 2006; Chu et al., 2010
*Solanum lycopersicon NAS (chln)*	NA synthesis	Cytosol/ Roots and shoots	Constitutive expression of *SlFRO*1 and *SlIRT1*	High Fe accumulation in shoots Higher Fe in older leaves vs. younger leaves	Foliar application of Fe or NA	Scholz et al., 1985; Garcia et al., 2013
*Unknown (brz)*	Unknown	Unknown	Constitutive expression of *PsFRO*1 and *PsIRT1*	High Fe accumulation in shoots Higher Fe in older leaves vs. younger leaves	Foliar application of Fe	Kneen et al., 1990; Garcia et al., 2013
Unknown (*dgl*)	Unknown	Unknown	Constitutive expression of *PsFRO*1 and *PsIRT1*	High Fe accumulation in older leaves	Grafting Wt shoots onto *dgl* roots	Grusak and Pezeshgi, 1996; Garcia et al., 2013

**FIGURE 2:**
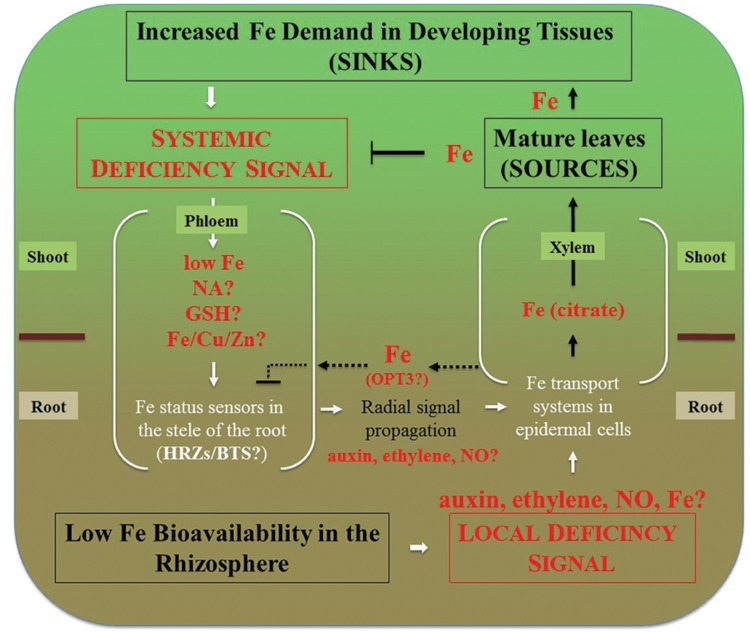
**Integration of local and systemic regulation of Fe deficiency responses in dicots.** Systemic signaling is thought to be initiated in sink tissues such as young leaves, developing reproductive organs and seeds. Signal is transmitted to roots via the phloem. Decreased concentration of Fe, or the ratio of Fe to other elements such as Zn, Cu, as well as NA itself have been considered to play a role in systemic signaling. While Fe has been considered to act as a repressive signal, the generation of a promotive signal that is propagated from shoots to roots via the phloem has been suggested as well (Enomoto and Goto, 2008). These signals are then percepted in the vasculature of roots and radially transmitted toward root epidermal cells that respond by increased expression of Fe uptake and root-to-shoot partitioning systems. Local signaling events are initiated by the fluctuation of Fe bioavailability in the rhizosphere and result in upregulation of Fe uptake and root-to-shoot partitioning transport systems. We note that Fe itself is essential for the expression of local Fe deficiency responses. For example, Vert et al. (2003) have shown that prolonged Fe deficiency stops expression of *IRT1* and *FRO2* in *A. thaliana* and this transcriptional response is restored by Fe supplementation of Fe-starved plants. Therefore, low Fe bioavailability in the rhizosphere depicts the situation where Fe concentration in the apoplast is sufficient to induce Fe deficiency responses. Fe delivery to sources (mature leaves) via xylem and subsequent Fe loading into the phloem downregulates systemic signaling and might override local Fe deficiency signaling. For example, the severely repressed lateral root elongation in response to local Fe supply in the *irt1* mutant of *A. thaliana* can be circumvented by the application of Fe to the shoot (Giehl et al., 2012). Dashed lines represent putative interactions between the xylem and the phloem in the root that might be mediated by OPT3 to fine-tune crosstalk between local and systemic signaling to prevent excessive Fe accumulation. Under Fe deficiency, roots increase the production of ethylene and NO that might serve in the radial propagation of the signal, as well as mediate local signaling.

Expression of OPT3 in roots is very low under standard condition but increases significantly under Fe deficiency (Zhai et al., 2014), raising a question of what is the role of OPT3-mediated xylem-to-phloem transfer in Fe-deficient roots. Given the repressive role of the phloem Fe on expression of Fe uptake genes in roots, it is possible that OPT3-mediated transfer of Fe from the xylem to the phloem is needed to fine-tune Fe deficiency responses to prevent excessive Fe uptake and partitioning to the shoot (**Figure 2**). It would be interesting to test if expression of *OPT*3 in the root of the *opt3* mutant would alter its transcriptional Fe deficiency responses. It is likely, however, that OPT3 function in the shoot in loading Fe into the phloem and partitioning into sinks is central to systemic Fe deficiency signaling.

Similar to *opt3* mutant alleles of *Arabidopsis, dgl* and *brz* mutants of pea accumulate more Fe in leaves, have increased expression of Fe acquisition genes, and a defect in remobilizing Fe from older to younger leaves (Kneen et al., 1990; Grusak and Pezeshgi, 1996; Garcia et al., 2013) and **Table 1**. Foliar application of Fe to the *dgl* mutant does not downregulate expression of *PsIRT1* and *PsFRO1*, suggesting that similar to *opt3* mutants of *A. thaliana*, the *dgl* mutant cannot move Fe or Fe-related signal into the phloem (Garcia et al., 2013). Due to the similarity of phenotypes, it is possible that *dgl* is an OPT3 ortholog. In contrast, application of Fe onto leaves of the *brz* mutant suppresses constitutive expression of *PsFRO2* and *PsIRT1*, suggesting that *brz* is able to move an Fe-related signal into the phloem (Garcia et al., 2013). Future studies on *dgl* and *brz* are needed to understand the contribution of these genes to shoot-to-root communication of Fe status.

Bashir et al. (2015) have recently characterized an OPT3 ortholog in *Oryza sativa*, OsOPT7. This group have found that similar to *AtOPT3, OsOPT7* is upregulated by Fe deficiency in both roots and shoots, and its translational product localizes to the plasma membrane. Further, the *opt7-1* mutant exhibits upregulated expression of Fe-deficiency responsive genes while hyperaccumulating Fe in shoots even under Fe replete conditions. Unlike *opt3* mutant alleles of *A. thaliana*, the constitutive transcriptional Fe-deficiency response of the *opt7-1* mutant was observed mainly in shoots, not roots. The authors suggested that foliar Fe in the *opt7-1* mutant is unavailable for the distribution, resulting in a local foliar Fe-deficiency response (Bashir et al., 2015). In contrast to AtOPT3, OsOPT7 seems to not to be involved in shoot-to-root communication of Fe status. Additionally, OsOPT7 does not functionally complement the *fet3fet4* Fe uptake mutant of *S. cerevisiae* and does not confer transport of Fe^2+^ or Fe(III)-NA or Fe(II) complexed with the phytosiderophore 2′-deoxymugineic acid (DMA) in *Xenopus* oocytes. Whether the function of OsOPT7 is compromised in the heterologous system or it transports other substrates, is yet to be determined.

### Crosstalk Between Different Elements in Iron Deficiency Signaling

The total metal composition of cells, the metallome, is very complex. Further, transition metals favor similar coordination geometries and ligand preferences (Haas and Franz, 2009). Therefore, among the overarching questions in the field is how the specificity and selectivity of metal sensing and signaling is achieved. Evidence from structural and biochemical studies of bacterial metal sensing systems have shown that selectivity and specificity of binding is determined by the metal binding properties of the ligand (such as affinity, measured as K*_d_*) and by the metal availability in the cytosol (metal access; Giedroc and Arunkumar, 2007; Waldron et al., 2009; Helmann, 2014). For example, since transition metals bind to their ligands with a K*_d_* that generally follows the Irving–Williams series (Pd > Cu > Ni > Co > Zn > Cd > Fe > Mn > Mg; Irving and Williams, 1953), equimolar concentrations of Fe, Cu, and Zn would result in selection of Cu and Zn for metal ligands and/or sensors over Fe. However, the situation *in vivo* is much more complex and the accessibility of elements is controlled by metal uptake and redistribution in subcellular compartments (Blaby-Haas and Merchant, 2014). As a result, concentrations of Cu and Zn in the symplast of plants are orders of magnitude lower than Fe (**Table 2**) explaining why strongLMW ligands for transition metals such as NA in non-graminaceous plants, is associated preferentially with Fe but not Cu or Zn in the symplast under standard growth conditions. However, a different scenario might occur under Fe deficiency, which alters the metallome of different species including plants. The most common metallome response of *A. thaliana* to Fe deficiency includes an increase in the concentrations of Mn, Co, Zn, and Cd and decrease in the concentration of Mo (Baxter et al., 2008). In addition, it has been shown that the concentration of Cu doubles in the rosette leaves of Fe-deficient *A. thaliana* within 24 h of switching plants to Fe-deficient nutrient solution and that optimal Cu supply is required for Fe deficiency responses (Bernal et al., 2012; Waters et al., 2012; Waters and Armbrust, 2013). It is noteworthy that concentration of Zn, Cu, Mn, and Co is significantly higher in shoots of the *opt3-2* and *opt3-3* mutants that exhibit constitutive Fe deficiency responses (Stacey et al., 2008; Zhai et al., 2014). Under these circumstances, Fe ligands and regulators might be mismetallated that, in turn, could be a contributing factor to Fe deficiency signaling.

**Table 2:** Estimated concentrations of Fe, Zn, and Cu in the cell and in the phloem sap and affinity constants (K*_d_*) for the formation of the indicated metal-ligand complexes.

	Fe^2+^	Zn^2+^	Cu^2+^
Cytoplasmic concentration (M)	10^-6^ Kruszewski, 2004	10^-11^; 10^-12^ Silva and Williams, 2001; Bozym et al., 2006; 0.4 × 10^-9^ Lanquar et al., 2014	10^-15^; <10^-18^ Silva and Williams, 2001; Kuchar and Hausinger, 2004
Phloem sap concentration (M)	1.7 × 10^-4^ Marschner, 1995	2.4 × 10^-4^ Marschner, 1995	1.9 × 10^-5^ Marschner, 1995
Xylem sap concentration (M)	10^-5^ Marschner, 1995	2 × 10^-5^ Marschner, 1995	1.7 × 10^-6^ Marschner, 1995
Log K*_d_* (NA)	12.1 Callahan et al., 2006	14.7/15.4 Callahan et al., 2006	18.6 Callahan et al., 2006
Log K*_d_* (GSH)	5.1 Hider and Kong, (2011)	12.5 Martell and Smith, 1974 for ML_2_/M.L^2^	19.9^∗^ Martell and Smith, 1974 for M(HL)_2_/M.(HL)^2^
Log K*_d_* (citrate)	4.4 Callahan et al., 2006	5.0 Callahan et al., 2006	5.9 Callahan et al., 2006

*We note that the precise concentration of kinetically labile elements in the plant cell has not been documented except for Zn (Lanquar et al., 2014). Data are compiled based on indicated reports and so these values may differ considerably between organisms, cells, and cellular compartments, and as a result of the different analytical methods and conditions that have been employed.*

*^∗^The stability constant was enumerated for Cu^+^.*

In bacterial systems, non-cognate metals might be discriminated against because the ability of some metalloregulators to exert transcriptional changes is also determined by allostery and thus, only the correct metal bound in an optimal conformation to the metalloregulator can alter its DNA-binding capacity to regulate gene expression (Giedroc and Arunkumar, 2007; Waldron et al., 2009; Helmann, 2014). Whether similar regulatory events occur in plants is not yet explored. Notably, a positive regulator of Fe-deficiency-responsive genes in rice, **I**ron **D**eficiency-responsive **E**lement-binding **F**actor **1** (IDEF1) that was recently identified as a putative Fe-sensor, binds not only ionized Fe but also other divalent metals such as Zn^2+^, Cu^2+^, and Ni^2+^ (Kobayashi et al., 2012). Whether IDEF1 regulates expression of its targets *via* allosteric association with DNA is unknown. Nevertheless, based on these findings and the fact that Fe deficiency combined with deficiencies of other divalent metals mitigate the symptoms of Fe deficiency in tobacco (Kobayashi et al., 2003), it has been proposed that plant sensors might detect the cellular concentration ratio between Fe and other metals rather than their absolute concentration (Kobayashi and Nishizawa, 2014). In this regard, **F**erric **U**ptake **R**egulator (Fur) in *Bacillus subtilis* senses Fe sufficiency, **M**a**n**ganese transport **R**egulator (MnR) senses Mn sufficiency, while a Fur homolog, PerR, senses the intracellular Fe/Mn ratio for the subsequent transcriptional regulation of their targets (Helmann, 2014). In addition to IDEF1, two more recently identified putative Fe sensors from rice and *A. thaliana*, E3 ubiquitin ligases HRZs/BTS were found to bind not only Fe but also Zn ions and negatively regulate Fe acquisition under conditions of Fe sufficiency in both graminaceous and non-graminaceous plants (Kobayashi et al., 2013; Kobayashi and Nishizawa, 2015; Selote et al., 2015). Selote et al. (2015) have shown that Fe binding destabilizes HRZ/BTS in *A. thaliana* and proposed that HRZ/BTS adjusts Fe deficiency responses, ensuring sufficient but not excess Fe uptake into roots.

## Iron Ligands as Putative Signals

Among the most recognized Fe ligands are citric acid, NA, and most recently GSH. These ligands have a wide range of affinities to Fe and other metals (**Table 2**), which determine their ligand exchange reactions and might influence Fe deficiency signaling.

### Citric Acid

Citric acid has been proposed to be a preferred Fe ligand under acidic conditions of the vacuole, the xylem sap and the apoplast (Rellan-Alvarez et al., 2010). The important role of citrate in Fe-deficiency signaling has been documented by the characterization of *frd3* mutants of *A. thaliana* with a non-functional citrate transporter gene (Rogers and Guerinot, 2002; Green and Rogers, 2004; Durrett et al., 2007) and **Table 1**. These mutants have 40% less citrate in the xylem than those of the wild-type, accumulate Fe in roots and shoots while manifesting the constitutive upregulation of *IRT1* and *FRO2* in roots (Rogers and Guerinot, 2002). Subsequent experiments have shown that the *frd3* mutation leads to the retention of Fe in the apoplast of xylem vessels spanning the root-to-leaf vasculature leading to decreased Fe concentration in the symplast of leaf mesophyll cells (Green and Rogers, 2004; Roschzttardtz et al., 2011). As a result of Fe mislocalization in the *frd3* mutant, leaf cells experience Fe deficiency and signal to the root to increase expression of Fe uptake genes. Foliar application of Fe decreases the expression of *IRT1* and *FRO2* in the root of the *frd3* mutant (Garcia et al., 2013) suggesting that a repressive Fe-related shoot-to-root signaling *via* the phloem is functional in these mutants. In addition to the indirect role of citrate in systemic Fe status signaling, citrate is regarded among the retrograde signals communicating changes in the metabolic status of mitochondria to the nucleus occurring under Fe deficiency (reviewed in, Vigani et al., 2013b).

### Nicotianamine

The amino acid derivative, NA, serves as a preferred Fe ligand in the cytosol including the shoot-to-root phloem symplast continuum since the neutral pH values in these tissues favor the formation and stability of the Fe–NA complex (von Wiren et al., 1999; Rellán-Álvarez et al., 2008). NA is synthesized from *S*-adenosylmethionine in a reaction catalyzed by NA synthase (NAS), encoded by four *NAS* genes in *A. thaliana* (Ling et al., 1999). The physiological and genetic studies of the quadruple *nas4x-1* and *nas4x-2* mutants of *A. thaliana* and the tomato *chln* mutant provide evidence that NA is needed for systemic Fe status signaling (**Table 1**). At the vegetative stage, rosette leaves of the *nas4x-1* mutant allele have a significantly lower concentration of NA and a higher concentration of Fe, along with minor leaf chlorosis that intensifies during the transition from vegetative to reproductive growth (Klatte et al., 2009). Additionally, *nas4x-1* mutants have elevated expression levels of *IRT1, FRO2*, and *FIT* (encoding **F**ER-like **i**ron-deficiency-induced bHLH **t**ranscription factor, Bauer et al., 2007) in roots under Fe-replete conditions (**Table 1** and Klatte et al., 2009). In contrast, the quadruple *nas4x-2* mutant that does not synthesize NA shows strong leaf chlorosis and is sterile (Schuler et al., 2012). Schuler et al. (2012) have shown that Fe accumulates in the phloem of the *nas4x-2* mutant and the chlorotic phenotype of young leaves of *nas4x-2* can be rescued by NA application onto the leaf surface. From these experiments, Schuler et al. (2012) proposed that NA functions in Fe mobilization out of the phloem to sink organs. Whether NA rescues the constitutive Fe deficiency response in roots of the *nas4x-2* mutant has not been tested. However, given the role of NA in maintaining Fe bioavailability in the shoot-to-root symplast continuum, the decreased NA concentration in leaves of the *nas4x-1* mutant and elevated expression of *IRT1, FRO2* and their regulator *FIT* in roots of plants grown under control conditions is consistent with the hypothesis that the decreased supply of bioavailable Fe into roots might be responsible for constitutive Fe deficiency responses of the root.

The tomato ***chl****oro****n****erva* (*chln*) mutant contains a defect in *NAS* as well (Ling et al., 1999) and similar to *nas4x-1* mutants, *chln* develops interveinal chlorosis, displays constitutive Fe uptake and hyperaccumulates Fe in shoots (Scholz et al., 1985; Pich and Scholz, 1996) and **Table 1**. The chlorotic phenotype of *chln* is rescued by foliar application of NA, which also decreases constitutive Fe uptake (Scholz et al., 1985; Garcia et al., 2013). This effect of NA on the regulation of Fe uptake systems in roots is likely due to the role of NA in the recirculation of bioavailable Fe from shoots to roots and by aiding in Fe remobilization out of phloem vessels in the root for subsequent signal propagation toward epidermal cells to control Fe uptake (**Figure 2**).

With regard to the direct involvement of NA in local and systemic Fe status signaling, it was reported that *frd3* mutants with impaired root-to-shoot translocation of Fe-citrate, manifest a twofold higher NA levels in roots and shoots than the wild-type (Rogers and Guerinot, 2002). Schuler et al. (2012) suggested that citrate and NA act, in part, redundantly in the long-distance transport of Fe from roots to leaves. This however, does not exclude the possibility that NA itself may also act as a shoot-to-root promotive signal reporting on the Fe status of the shoot. It is noteworthy that given the complexity of the metallome of the phloem sap and K*_d_* of NA for Cu^2+^ and Zn^2+^ (**Table 2**), it is unlikely that NA exists in the “apo” form in the *frd3* plants. It is possible that recirculating NA complexed with Cu or Zn, delivers latter metals instead of Fe to metalloregulators, altering their effects on the expression of Fe uptake genes in the root of the *frd3* plants (see Crosstalk between Different Elements in Iron Deficiency Signaling).

Nicotianamine might not be the only ligand in the phloem involved in the shoot-to-root communication of Fe status. For example, the double *ysl1ysl3* mutant of *A. thaliana* lacks phloem-associated metal-NA transporters and has a decreased concentration of Fe in roots and shoots (Waters et al., 2006). However, the long-distance signaling is not altered in the *ysl1ysl3* mutant: its exhibits a wild-type level of *IRT1* and *FRO2* expression unless is grown under Fe-deficient conditions. Further, the suppression of Fe deficiency responses in roots of the *chln* mutant by foliar application of Fe (Scholz et al., 1985; Garcia et al., 2013) points to the existence of other Fe ligand(s) that contribute to Fe solubility/bioavailability in the phloem resulting in the ability of Fe to exert its suppressive role. In this regard, a protein capable of binding to Fe, **i**ron **t**ransporter **p**rotein (ITP), was found in the phloem sap of *R. communis* (Krüger et al., 2002), and most recently, a metallothionein-like protein type 2B, and a low-molecular weight protein with similarity to a phloem-specific protein from *Medicago truncatula*, were identified associated with Fe in the phloem sap of *Lupinus texensis* (Lattanzio et al., 2013). However, their contribution to Fe homeostasis and signaling has not been yet examined.

### Glutathione

The ubiquitous tripeptide glutathione (γ-glutamyl-cysteinyl-glycine, GSH) is an essential monothiol that is present in millimolar concentrations in the cell and is the major determinant of the cellular redox status in addition to its recognized function in the regulation of cell proliferation and xenobiotic detoxification (Noctor et al., 2012; Schnaubelt et al., 2015). Most recent studies in *Saccharomyces cerevisiae* and *Schizosaccharomyces pombe* uncovered the involvement of GSH in Fe metabolism and signaling through its role in the maturation of extra-mitochondrial Fe–S clusters (Sipos et al., 2002; Mühlenhoff et al., 2010; Hider and Kong, 2011, 2013; Kumar et al., 2011). Sipos et al. (2002) have shown that deletion of the gene encoding the enzyme that catalyzes the first step of GSH biosynthesis, *GSH1* (*Δgsh1*) leads to the growth arrest, the accumulation of high Fe concentrations in the mitochondria and the decrease in the maturation of Fe–S proteins in the cytosol. GSH is suggested to protect Fe–S clusters in small oxidoreductases, glutaredoxins (GRXs), that facilitate Fe–S cluster biosynthesis (Bandyopadhyay et al., 2008). Kumar et al. (2011) have shown that low or high GSH levels trigger an intense Fe starvation-like response in yeast cells and impair the activity of extra-mitochondrial Fe–S cluster containing enzymes. Further, these authors emphasized the role of GSH in Fe metabolism rather than in maintaining the redox state of yeast cells. Hider and Kong (2011) used speciation plots that consider typical cellular concentrations of potential Fe (II) ligands in the cytoplasm and stability constants for Fe(II) complexes to show that GSH dominates over other potential Fe ligands, such as citrate in the cytoplasm of yeast cells. These authors proposed that Fe (II)-GSH is a dominant cytoplasmic ferrous Fe pool in yeast. Furthermore, they suggested that association of Fe with GSH provides selectivity over other transition elements in providing substrate for Fe–S cluster assembly (Hider and Kong, 2011).

The role of GSH in Fe homeostasis and signaling in plants has not been comprehensively addressed but it has been shown that GSH is also important to maintain proper homeostasis and crosstalk between Zn and Fe metabolism in *A. thaliana* (Shanmugam et al., 2012). It is noteworthy, however, that role of GSH in Fe homeostasis in plants might be somewhat different from yeast, bacteria, and mammals because, unlike these organisms, plants synthesize NA, which K*_d_* for Fe(II) is orders of magnitude higher than that of Fe(II)-GSH (**Table 2**). Therefore, NA might dominate over GSH in the cytoplasm in plants. However, a different scenario might occur in subcellular compartments, particularly in mitochondria and chloroplasts both bearing high Fe and both responsible for Fe–S cluster and heme synthesis (Balk and Schaedler, 2014). Furthermore, the first rate-limited step in GSH synthesis, namely formation of γ-EC, occurs exclusively in chloroplasts due to exclusive chloroplast localization of the first enzyme in GSH production, glutamate cysteine ligase (GSH1), and homozygous mutation in *GSH1* is lethal (Wachter et al., 2005; Cairns et al., 2006). The second enzyme glutathione synthetase (GSH2) is located in both the cytosol and plastids and cytosolic form can rescue GSH1 lethality suggesting the translocation of γ-EC and GSH in and out of chloroplast (Pasternak et al., 2008). *In vitro* studies have shown that GSH is required for Fe–S cluster assembly using chloroplast GrxS14 from poplar (Bandyopadhyay et al., 2008). However, much more studies are needed to shed light on the role of GSH in Fe homeostasis and signaling in these subcellular compartments.

With regard to the role of GSH in long-distance Fe signaling and homeostasis, Mendoza-Cozatl et al. (2014) have tested whether foliar application of GSH or Fe-glutathione complex would rescue the constitutive high activity of the root ferric reductase in the *opt3-2* mutant. However, in all cases the activity of the root ferric reductase remained constitutively high in *opt3-2* vs. wild-type, suggesting that shoot-to-root transport of GSH alone has little effect on the long-distance signaling of the Fe status at least with regard to OPT3 function in *Arabidopsis*. It is noteworthy, however, that we found that overaccumulation of Fe in leaves of the *opt3-3* mutant is associated with twofold increase in the concentration of GSH compared to wild-type, both grown under Fe-sufficient conditions (Zhai, 2011). This suggests that GSH might be involved in buffering Fe concentration in leaf cells of the *opt3* mutant.

## Concluding Remarks

Despite significant progress in our understanding of the regulation of Fe homeostasis in plants, the exact nature of local and systemic Fe signals, their interactions with sensors in different tissues and cell types as well as the signal propagation from the vascular cylinder in roots to root epidermal cells to trigger Fe deficiency response, remain elusive. Here we discussed the current status of knowledge of the contribution of Fe, transition metal ligands and interactions between metals in local and systemic Fe signaling. From the current outlook, studies of the ligand environment of Fe, its distribution among different cellular compartments, the composition and metal interactions of potential Fe ligands in the phloem sap in Fe-signaling mutants will expand our knowledge of how Fe signaling is achieved.

Fe-sensors, HRZs/BTS, which are transcriptionally upregulated by Fe deficiency in root pericycle in *A. thaliana* and in the stele in rice (Long et al., 2010; Kobayashi et al., 2013) might be involved in the perception of the phloem-derived Fe signal. In addition, plant hormones, auxin, ethylene, abscisic acid, gibberellin, brassinosteroids, and nitric oxide, not discussed here, have been reported to act as positive regulators of Fe deficiency (reviewed in, Hindt and Guerinot, 2012; Wang et al., 2012; Kobayashi and Nishizawa, 2014). Of these, ethylene and NO, which level increases in roots under Fe deficiency are among the plausible candidates of radial signal propagation from the vasculature to the root epidermal cells (**Figure 2**). It has been shown that exogenous application of 1-Aminocyclopropane-1-carboxylic acid (ACC, ethylene precursor) or *S*-nitrosoglutathione (GSNO, NO donor) to Fe-deficient plants up-regulates the expression of Fe-acquisition genes, but this effect does not occur in Fe-deficient plants sprayed with Fe (Garcia et al., 2013), suggesting that ethylene and NO act downstream of the phloem-derived repressive Fe signal.

Another important area deals with metabolic adjustments and crosstalk between mitochondria and chloroplast caused by changes in Fe bioavailability. Fe is required for heme and Fe–S clusters synthesis with both processes occurring in mitochondria and chloroplasts. Therefore, both compartments compete for labile Fe pools and are implicated in the retrograde Fe deficiency signaling (Vigani et al., 2013a,b). However, we are still far from understanding the nature of mitochondria- and chloroplast-derived signals and how these signals are integrated to maintain Fe status of plants. Further studies of the signaling mechanisms that plants use to coordinate Fe demand with Fe uptake, transport and tissue partitioning will facilitate molecular breeding efforts directed on improving crop yield on marginal soils and increasing Fe concentration in edible plant organs to improve human nutrition and health.

## Conflict of Interest Statement

The authors declare that the research was conducted in the absence of any commercial or financial relationships that could be construed as a potential conflict of interest.
